# Editorial

**Published:** 1913-12

**Authors:** 


### EDITORIAL

The Business Office of the *Journal-Record* is 23 West Alabama Street.

The Editorial Office is 1014-15 Atlanta National Bank Building.

Address all Business Communications to *Journal-Record of Medicine*, 23 West Alabama Street.

Make remittances payable to *The Journal-Record of Medicine.*

On matters pertaining to the Editorial and Original Communications, address Edgar G. Ballenger, M. D., Atlanta, Ga.

Reprints of Original Articles will be furnished at cost price. Requests for the same should always be made in THE MANUSCRIPT.

We will present, postpaid, on request to each contributor of an original article, twenty (20) marked copies of *The Journal-Record of Medicine* containing such article.

### GEORGIA SURGEONS' CLUB.

As forecasted in a previous number of the Journal-Record the Georgia Surgeons' Club held its initial meeting in Atlanta, November the 16th and 17th. An interesting program was planned and executed. There was a large number of visiting physicians attending the clinics and we feel safe in predicting an even larger number next year. An unusually pleasant banquet was given Wednesday night, at which there was a large crowd of Atlanta and visiting physicians and surgeons. Plans were instituted to arrange visits to New Orleans in February, and later to Baltimore, Philadelphia, New York and other large cities. Further details will be published as the plans are consummated.

All of the officers were re-elected as follows: E. C. Davis of Atlanta, president; Thomas J. McArthur, of Cordele, vice-president, and R. M. Harbin, of Rome, secretary and treasurer. The executive committee consists of W. S. Goldsmith chairman; G. R. White, of Savannah, and W. W. Battey, Jr., of Rome.

---

### EARLY DIAGNOSIS.

When children are attacked with the eruptive fevers, it is often impossible to say at once just what the trouble is. The rash or some other distinctive symptom must appear before decision is made. The family as well as the physician understands this and all concerned think nothing of the delay. The unfortunate thing about it is that one is likely to get the habit of waiting a while till something striking develops and the disease, whatever it may be, gets well underway in its course. Primarily this is due to ignorance. There are some of the exanthemata that no one can decide about at first because no one knows. There can be no blame attached to this, for we are all striving to find the unknown, and it is not anyone's fault that it has not yet been discovered.

In Diphtheria, we have the means of early diagnosis and every community, almost, provides its physicians with laboratory tests of the pharyngeal secretions. The early diagnosis and the possibility of prompt treatment have saved a tremendous percentage of lives, as we all know.

But there are other diseases that we can no better afford to wait for than we could in diphtheria. Notable among these is Acute Osteomyelitis. It is a disease that is obscured from us because of the term "rheumatism" and the control the word has over the lay and sometimes the professional mind. When the child begins to suffer pains in the long bones and at the joint, the family is likely to delay a day or two while they decide what kind of rheumatism it is and whether it can be outgrown. When the doctor is called in, precious minutes have been lost, for the progress is often so rapid as to make invasion of the shafts seem almost a matter of minutes in its extension. Certainly there should be no delay in diagnosis nor in the radical surgical treatment the disease demands. The bone should be freed from pus instantly, both under the periosteum and in the medullary canal if one will prevent the chronic osteomyelitis from ensuing. Cripple legs often show the lack of early diagnosis of acute osteomyelitis.

The early analysis of the conditions causing club feet of various sorts will prevent the use of needless and crude apparatus to push or force them into shape. Not only is a child's gait interfered with by club feet, but the development of his whole moral nature is profoundly affected by the limitation of childish activities and moral companionships the crippling entails. An early study of these conditions and the application of correct mechanical principles in surgery have been conclusively proved by photographs, skiagraphs and moving pictures to be successful in practically curing the deformities. Here again, while the pressure for time it not so great as in the previous instances, there is a serious loss in the developmental power of the component parts of the deformed limb as the years are allowed to pass.

X-Ray pictures are of the utmost value in assisting the study of the foregoing, and in addition they have fields of their own for the most enlightening investigations. This is shown in the gastrointestinal work that is being done by advanced radiographers. No longer need the physician speculate upon the result of his palpations, splash sounds and pressure symptoms while trying to decide upon intra abdominal conditions. He can have photographs made and skilled interpretations of them given that will show beyond a peradventure displacements and enlargements of the viscera, and in addition, early diagnosis can be made of cancer and other troubles that will bring them within the time limit of curative surgery.

The scientist of to-day is concerning himself with the little things that show the beginnings of the gross lesions, and by learning to recognize them the instant they appear, he prevents destructive diseases and saves health and lives.

